# Strip thickness prediction method based on improved border collie optimizing LSTM

**DOI:** 10.7717/peerj-cs.1114

**Published:** 2022-10-25

**Authors:** Lijie Sun, Lin Zeng, Hongjuan Zhou, Lei Zhang

**Affiliations:** 1 School of Electronics and Information Engineering, Taizhou University, Taizhou, Zhejiang, China; 2 State Grid Taizhou Power Supply Company, Taizhou, Zhejiang, China; 3 China Water Resources & Hydropower Engineering Bohai Consultancy Co. Ltd., Tianjin, China; 4 School of Information, Liaoning University, Shenyang, Liaoning, China

**Keywords:** Strip thickness, Mutual information, Feature selection, Border Collie, LSTM

## Abstract

**Background:**

The thickness accuracy of strip is an important indicator to measure the quality of strip, and the control of the thickness accuracy of strip is the key for the high-quality strip products in the rolling industry.

**Methods:**

A thickness prediction method of strip based on Long Short-Term Memory (LSTM) optimized by improved border collie optimization (IBCO) algorithm is proposed. First, chaotic mapping and dynamic weighting strategy are introduced into IBCO to overcome the shortcomings of uneven initial population distribution and inaccurate optimization states of some individuals in Border Collie Optimization (BCO). Second, Long Short-Term Memory (LSTM) which can effectively deal with time series data and alleviate long-term dependencies is adopted. What’s more, IBCO is utilized to optimize parameters to mitigate the influence of hyperparameters such as the number of hidden neurons and learning rate on the prediction accuracy of LSTM, so IBCO-LSTM is established.

**Results:**

The experiments are carried out on the measured strip data, which proves the excellent prediction performance of IBCO-LSTM. The experiments are carried out on the actual strip data, which prove that IBCO-LSTM has excellent capability of prediction.

## Introduction

Many areas of industrial production are closely related to the steel industry. With the rapid development of various industrial technologies, the industries that use strip steel as a raw material for production have higher requirements for the quality of finished products, so the requirements for the quality of the strip rolled by the iron industry are also increasing. The key to improving the strip quality is to improve the strip thickness accuracy; therefore, more and more scholars regard the prediction of strip thickness as an important research topic (Ding et al., 2013).

At present, the method of improving the strip thickness prediction accuracy through mathematical models has become an important technology to promote the development and progress of steel rolling technology. The strip thickness prediction model is to express the variables involved in the strip thickness rolling process and the relationship between them through mathematics, and to control the process on this basis. With the comprehensive application of machine learning in industrial production, more and more strip thickness prediction models with neural network as the core have become popular (Maazoun et al., 2022; Ganesh & Ramachandra Murthy, 2021). Ortmann (1994) took the lead in using neural networks to develop prediction models for parameters such as roll width, surface temperature and rolling force, which greatly improved the prediction accuracy.

Rao & Mohan Emani (2001) established the prediction model based on neural network for metal deformation resistance during hot rolling. Wang, Chang & Hua (2012) proposed a method based on BP neural network with prediction of hot rolling aluminum transverse thickness according to multi-channel measurement characteristics of IMS transverse thickness collection, which predicted every channel thickness accurately and obtained the transverse thickness distribution of aluminum strip. Mao et al. (2020) adopted BP neural network to model and predict the thickness of hot-dip galvanized zinc layer. Also, a genetic algorithm was introduced to optimize the BP neural network, and the initialization weights and biases of BP neural network were optimized in advance, which contributed to improve prediction accuracy and convergence ability of GA-BP.

However, the traditional shallow machine learning method has certain defects, such as the model is easy to fall into local optimum, difficult to extract deep features of data and easy to overfit (Brito, Susto & Brito, 2022; Li et al., 2020b; Ganesh & Murthy Ramachandra, 2021; Shafiq et al., 2020a; Tian et al., 2019). In contrast, the abilities of generalization and anti-noise of the model are weak. The deficiencies above limit the prediction accuracy of shallow learning model. Yet the deep learning models possess strong abilities of robustness, generalization, extraction of deep features and complex mapping relationship, which are more suitable for realizing accurate prediction of strip thickness (Wang et al., 2021; Kuźnar & Augustyn, 2021; Jiao, Peng & Dong, 2021; Xu et al., 2020; Li et al., 2020a).

Currently, deep learning methods, such as Deep Belief Network (DBN), Convolutional Neural Network (CNN) and Long Short-Term Memory (LSTM), have been used by many scholars to realize regression prediction (Sezer, Berat & Ahmet, 2020; Shafiq et al., 2020b; Tian et al., 2020; Yu et al., 2019b; Yu et al., 2019a). LSTM is a special recurrent neural network that transmits information of network state through gating mechanism to realize network memory function, which can alleviate not only the problem of long-range dependencies, but also the gradient disappearance and gradient explosion (Hou et al., 2021). In addition, LSTM is good at mining dependencies between nonlinear sequence data and time, and it is currently widely utilized in many fields such as sequence prediction, speech recognition, and machine translation (Zhang & Wang, 2022). Therefore, LSTM, as an effective model for learning long-term dependent features, is very suitable for dealing with the prediction problem of time series and widely utilized in various fields. Mohd et al. proposed a new method combining Laplace Scoring (LS), stochastic search optimization, and LSTM to accurately analyze the remaining service life of mechanical systems. The method was tested on the IMS bearing data set, and the experimental results showed that the prediction accuracy was significantly improved compared to other methods for predicting the remaining service life of bearings (Saufi & Hassan, 2021). Afrin & Yodo (2022) proposed an LSTM-CTP framework for predicting correlated traffic data based on LSTM, which performed spatio-temporal trend prediction on two different real-time traffic data sets and obtained better prediction performance. Ponnoprat (2021) proposed a effective seasonally-integrated autoencoder (SSAE) for short-term daily precipitation prediction. However, there are few studies related to LSTM in strip thickness prediction. Therefore, it is very promising to establish a strip thickness prediction model based on LSTM to better control strip thickness and strip quality.

The accuracy of the prediction model based on deep learning is affected by key super parameters, such as the number of hidden layer neurons and the learning rate. In order to avoid the reliance on manual experience for the parameters of the network structure, the method of finding the optimal parameters of the network structure through the swarm intelligence optimization algorithm is quite popular. Ahmet et al. used Genetic Algorithm (GA) to optimize parameters of LSTM and proposed GA-LSTM multi-step prediction model for influenza outbreak. Then, the experiments shown that the prediction effect of this model is better than that of other traditional models such as SVM (Kara, 2021; Yang, Yu & Lu, 2020). Beiranvand & Rajaee (2022) used Back Propagation Neural Network(BPNN) optimized by Lion Swarm Optimization (LSO) algorithm to predict the uniaxial compressive strength (UCS) of a novel rubber-sand concrete (RSC) material. The experiments were performed on data sets from RSC lab, which showed that LSO-BPNN possesses excellent ability of prediction. Li et al. (2022) proposed a hybrid approach that simultaneously considers the Variational Mode Decomposition (VMD) algorithm, the Particle Swarm Optimization (PSO) method and Bidirectional, Long Short-term Memory (Bi-LSTM). The results showed that the proposed PSO-VMD-Bi-LSTM has strong robustness for making uncertainty predictions and can be utilized to predict the typhoon speed (Li et al., 2022).

Based on the related researches from domestic and foreign countries, this article proposes a new prediction method of strip thickness based on Long Short-Term Memory (LSTM) optimized by improved border collie optimization (IBCO) algorithm. First, to enhance the uniformity and ergodicity of the population distribution, the chaotic mapping is introduced to Border Collie Optimization (BCO) algorithm to optimize the population initialization. Second, the method of mutual information is introduced to perform feature selection on the original strip steel data set, and the extracted important factors are formed into a new feature data set. Finally, according to the principle of mutual information feature extraction, the factors such as rolling speed, roll slit, mill current and rolling force are selected to form a multi-feature data set. Then, an LSTM whose hyperparameters are optimized by IBCO, namely IBCO-LSTM, is utilized to conduct experiments on the multi-feature data set, and the results indicate the excellent capability of prediction of the proposed method.

## Related Work

### Border collie optimization

Inspired by the herding behavior of border collies in daily life, Tulika Dutta et al. proposed Border Collie Optimization (BCO) algorithm (Dutta et al., 2021). Three border collies are randomly initialized: the lead dog, the left guide dog, and the right guide dog. The fitness values are *fit*_*f*_, *fit*_*le*_ and *fit*_*ri*_ respectively. The rest of the population consists of sheep, and the fitness value is denoted as *fit*_*s*_. The updates of the velocity of the three guide dogs at time *t* +1 are shown in Eq. (1):

(1) \begin{eqnarray*}{V}_{f,ri,le}(t+1)=\sqrt{{V}_{f,ri,le}(t)^{2}+2\times Ac{c}_{f,ri,le}(t)\times Po{p}_{f,ri,le}(t)}\end{eqnarray*}

where *Acc* (*t*) represents the acceleration of the three dogs at moment *t*, and *Pop* (*t*) represents their position at moment *t*. The update of the velocity of the aggregated sheep is shown in Eq. (2):

(2) \begin{eqnarray*}{V}_{sg}(t+1)=\sqrt{{V}_{f}(t+1)^{2}+2\times Ac{c}_{f}(t)\times Po{p}_{sg}(t)}.\end{eqnarray*}

Three guide dogs control the global search of the entire algorithm. They move in different directions and are independent of each other. They can quickly find regions in the large search space where optimal solutions are likely to exist. The movement of the flocks are influenced by the three guide dogs. Also, the flocks can focus on local searches in the space, and strive to find a better position. The updates of positions of the three border collies and the flock are shown in Eqs. (3) and (4).

\begin{eqnarray*}Po{p}_{f,ri,le}(t+1)& & ={V}_{f,ri,le}(t+1)\times Tim{e}_{f,ri,le}(t+1) \end{eqnarray*}

(3) \begin{eqnarray*}& & + \frac{1}{2} Ac{c}_{f,ri,le}(t+1)\times Tim{e}_{f,ri,le}(t+1)^{2}\end{eqnarray*}

\begin{eqnarray*}Po{p}_{sg}(t+1)& & ={V}_{sg}(t+1)\times Tim{e}_{sg}(t+1) \end{eqnarray*}

(4) \begin{eqnarray*}& & + \frac{1}{2} Ac{c}_{sg}(t+1)\times Tim{e}_{sg}(t+1)^{2}.\end{eqnarray*}

### LSTM

LSTM optimizes the hidden layer structure based on memory information like the recurrent neural network, and introduces a “gate” structure into the hidden layer neurons, namely input gate, forget gate and output gate, which control the update of historical data (Zhai et al., 2021).

Forget gate: The forget gate is responsible for selectively forgetting the state information transmitted in the previous moment, namely, forgetting the redundant information. The calculation process of the forget gate is shown in Eq. (5):

(5) \begin{eqnarray*}{f}_{t}=\sigma ({W}_{f}\cdot [{h}_{t-1},{x}_{t}]+{b}_{f})\end{eqnarray*}

where *f*_*t*_ is the output of the forget gate. *h*_*t*−__1_ is the hidden state at moment *t*-1. *x*_*t*_ is the input at moment *t*. *W*_*f*_ represents the weight matrix of the forget gate. *b*_*f*_ denotes the bias of the forget gate. *σ* represents the sigmoid activation function.

Input gate: The input gate determines the extent to which the current input information *x*_*t*_ is stored in the long-term state *C*_*t*_ and it controls which new information is added to the unit state *C*_*t*_. The calculation process of the input gate is shown in Eqs. (6) to (8):

(6) \begin{eqnarray*}{i}_{t}& & =\sigma ({W}_{i}\cdot [{h}_{t-1},{x}_{t}]+{b}_{i})\end{eqnarray*}

(7) \begin{eqnarray*}{\bar {C}}_{t}& & =\tanh \nolimits ({W}_{c}\cdot [{h}_{t-1},{x}_{t}]+{b}_{c})\end{eqnarray*}

(8) \begin{eqnarray*}{C}_{t}& & ={f}_{t}\times {C}_{t-1}+{i}_{t}\times {\bar {C}}_{t}\end{eqnarray*}

where *i*_*t*_ represents the output of the sigmoid activation function in the input gate. ${\bar {C}}_{t}$ denotes the candidate input. *C*_*t*_ is the unit state at time *t*.

Output gate: The output gate is responsible for determining which values the memory unit outputs at the current moment, namely, calculating the output based on the unit state. The calculation process of the output gate is shown in Eqs. (9) and (10):

(9) \begin{eqnarray*}{o}_{t}& & =\sigma ({W}_{o}\cdot [{h}_{t-1},{x}_{t}]+{b}_{o})\end{eqnarray*}

(10) \begin{eqnarray*}{h}_{t}& & ={o}_{t}\times \tanh \nolimits ({C}_{t})\end{eqnarray*}

where *o*_*t*_ is the output gate; *h*_*t*_ represents the hidden state of the memory unit at moment *t*.

### The proposed method

#### IBCO

##### (1) Population initialization method based on Tent chaotic mapping.

The border collie optimization (BCO) algorithm utilizes the randomly generated data as the initial population information, which will cause uneven distribution of individuals in the initial population, reduce the diversity of the population, seriously affect the efficiency of the algorithm in searching for the optimal solution, and even lead to the failure of the algorithm optimization. In order to enhance the uniformity and ergodicity of population distribution, chaotic mapping is introduced to optimize the population initialization. The chaotic mappings are generated by iterations of the deterministic nonlinear difference equation. The motion orbit is disordered, but its internal evolution is regular and can traverse the state space (Zhang et al., 2022).

At present, the widely used chaotic map is Logistic chaotic map, but some scholars have proved that Tent map has better ergodicity, uniformity and faster iteration speed than Logistic map (Zhang et al., 2021). Therefore, Tent mapping is quoted to BCO in this article, which is named improved BCO and denotes as IBCO. Tent mapping expression is shown in Eq. (11).

(11) \begin{eqnarray*}{x}_{n+1}= \left\{ \begin{array}{@{}ll@{}} \displaystyle \frac{{x}_{n}}{\alpha } ,&\displaystyle 0\leq {x}_{n}\leq \alpha \\ \displaystyle \frac{1-{x}_{n}}{1-\alpha } ,&\displaystyle \alpha \lt {x}_{n}\leq 1 \end{array} \right. \end{eqnarray*}

where *α* is the mapping parameter, the system is in a chaotic state when *α* is between 0 and 1, and the mapping chaos is very strong when *α*>0.43. In this article, take Tent mapping with *α* = 0.5, namely its most classical state, and the chaotic sequence obtained by the mapping is uniformly distributed. Tent mapping not only preserves the randomness of initialized individuals, but also improves the diversity of the population and the quality of the distribution of the search space, which makes the algorithm easy to jump away from the local optimal solution when solving function optimization problems and improves the global search capability.

**Figure 1:**
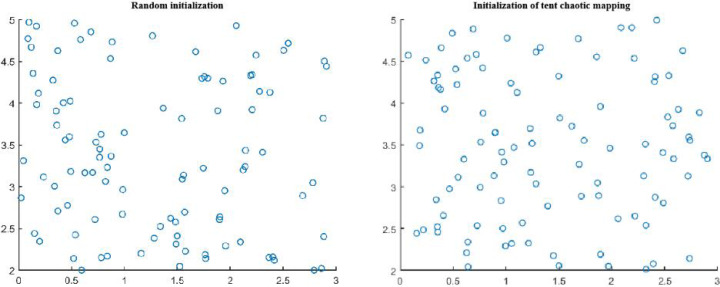
Comparison of population distribution initialization by random method and Tent chaotic mapping.

In order to verify the effectiveness of the initial population distribution in Tent chaotic map optimization algorithm, 100 points were randomly generated in the two-dimensional plane to conduct the initial population distribution experiment, as shown in Fig. 1.

It can be clearly seen from Fig. 1 that the uniformity and ergodicity of the initial population distribution in the border collie optimization (BCO) algorithm based on Tent chaotic mapping are significantly better than those of the algorithm population initialized by random method, which is more conducive to improving the global search ability of the algorithm.

##### (2) The update method of lock speed based on dynamic weighting strategy.

The original BCO algorithm does not reflect the speed effect of the guide dog with good fitness on flock speed, resulting in the inaccurate motion state of the tracked sheep and reducing the local optimization accuracy. Therefore, the weighted strategy of dynamic proportion is proposed to update the speed of the tracked sheep. The dynamic proportional weight can clearly show the importance of the left and right guide dogs after each iteration, so that the guide dogs with better position play a more important leading role in the flock, and more accurately guide the tracked sheep to move in the right direction. The dynamic weight of left and right guide dog speed is shown in Eqs. (12) and (13), and the improved method of speed of tracked sheep is shown in Eq. (14).

(12) \begin{eqnarray*}{\omega }_{le}& & = \frac{fi{t}_{ri}}{fi{t}_{le}+fi{t}_{ri}} \end{eqnarray*}

(13) \begin{eqnarray*}{\omega }_{ri}& & = \frac{fi{t}_{le}}{fi{t}_{le}+fi{t}_{ri}} \end{eqnarray*}

(14) \begin{eqnarray*}Vss& & = \frac{{\omega }_{le}{V}_{le}+{\omega }_{ri}{V}_{ri}}{({\omega }_{le}+{\omega }_{ri})} \end{eqnarray*}

where *ω*_*le*_ is the speed weight of the left guide dog; *ω*_*ri*_ is the speed weight of the right guided dog.

### LSTM optimized by IBCO

**Figure 2:**
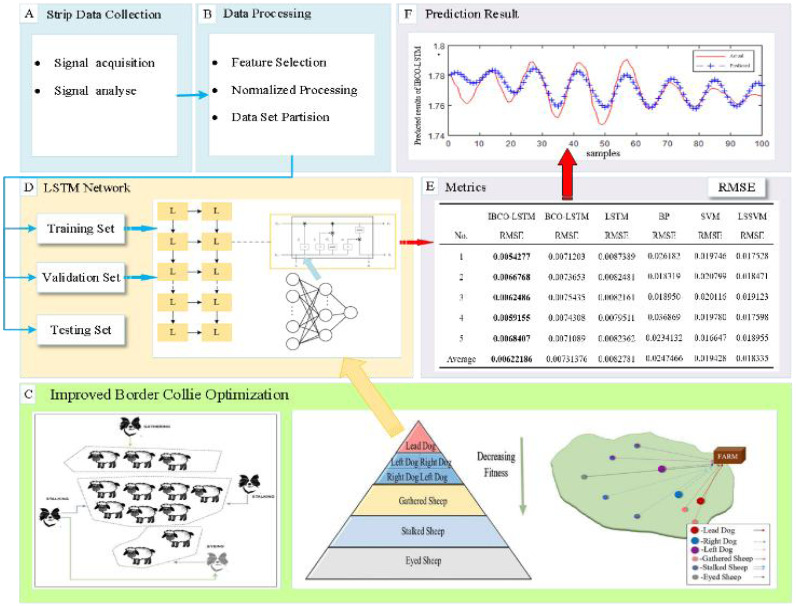
Overall flow chart of IBCO-LSTM strip thickness prediction model.

The important parameters of LSTM, namely the number of neurons of hidden layer and the learning rate, are difficult to determine and are often based on personal experience, which is random and can cause the prediction performance of the model to be very unstable. Therefore, IBCO-LSTM strip thickness prediction model is proposed, and the parameters of LSTM are optimized by using the improved BCO algorithm. The overall flow chart of IBCO-LSTM strip thickness prediction model is shown in Fig. 2.

 The optimal individual position (the position of the leading dog) in the algorithm is taken as the number and learning rate of neurons in the hidden layers of LSTM to establish the optimal prediction model. The optimization process of LSTM parameters by IBCO algorithm is as follows:

(1) Specify the basic parameters in the optimization process of parameters LSTM, including the size of the algorithm population n, the maximum number of iterations *Max_iterations*. Limit the neurons number of hidden layer and the search range of learning rate. And randomly initialize the velocity, acceleration and time of individual movements in the population.
(2) Initialize the population position of BCO algorithm by Tent chaotic mapping, and the individual positions *p*_1_, *p*_2_, *p*_3_ are set as the number of neurons and learning rate of each hidden layer of the model.
(3) Train the model by tenfold cross validation method, and the root mean square error between the actual thickness and the predicted thickness is used as the fitness function of IBCO.
(4) Carry out the iteration to calculate the position of individuals in each iteration and calculate the fitness value. The optimal historical position of individuals is determined by comparison and then the optimal position of the population is determined. When the maximum number of iterations is reached, the optimal individual position (the position of leader dog) is mapped to the number of neurons in the hidden layer of LSTM and the learning rate, and end the optimization process.

## Experiment and Result Analysis

### Data collection and analysis

**Figure 3:**
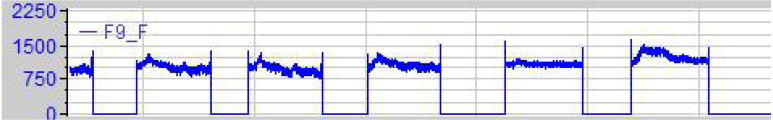
Rolling force signal diagram.

**Figure 4:**
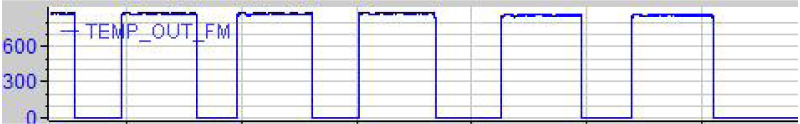
Plate and strip exit temperature signal diagram.

**Figure 5:**
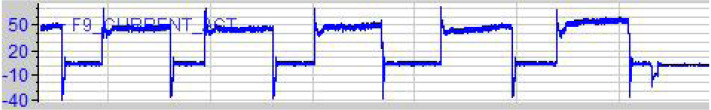
Rolling mill current signal diagram.

**Figure 6:**
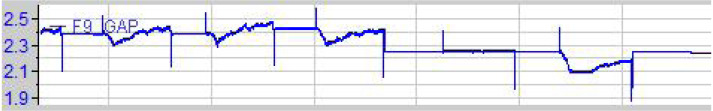
Rolling gap signal diagram.

**Figure 7:**
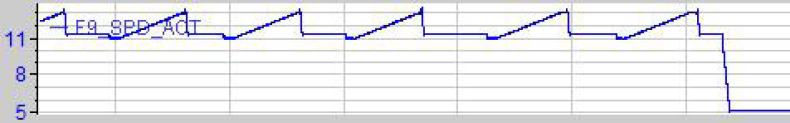
Rolling speed signal diagram.

**Figure 8:**
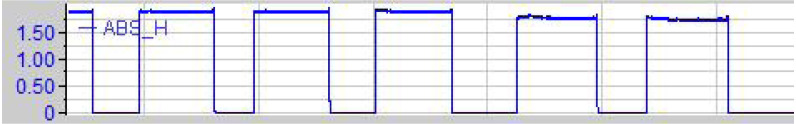
Strip thickness signal diagram.

In order to validate the performance of the proposed method, diverse experimental studies will be carried out. The experimental data are from the hot continuous rolling and finishing mill of a steel plant of a domestic iron and steel group, which includes nine stands. When collecting data, a thickness gauge is installed in the finishing mill to measure the thickness of the strip outlet. At the same time, sensors are installed on the hydraulic lower devices of nine flat roller stands in the finishing mill to collect the factors of the strip thickness. The strip thickness of the final collected data is numerical data, while the factor data exists in the form of signals, mainly including mill rolling force, strip outlet temperature, mill current, SONY value, rolling speed, and roll gap. Since the signal is complex and cannot be directly utilized as the experimental data, for this reason ibaAnalyzer is used to analyze the data and convert the signal data into numerical type sequences. After the analysis is completed, the multi-dimensional factor sequence and thickness are combined to form the original numerical strip data. Some factors are shown in Figs. 3 to 8.

### Rolling feature selection

The original strip data is actually a nonlinear time series, which contains many factors affecting the accuracy of strip thickness, but the influence degree of each factor is not the same, and there may be redundant factors with weak influence. The weak correlation redundancy factors should be eliminated, and the important factors with strong representativeness should be used as the input data characteristics of the model training, which can reduce the complexity of the model, shorten the training time and improve the generalization ability of the model.

Mutual information is usually used as an effective standard to measure the correlation between two random variables. This measurement is not only applicable to linear correlation variables, but also applicable to nonlinear correlation variables (Vergara Jorge & Estévez, 2014). It is a feature selection method widely used in the field of machine learning (Zhang & Wang, 2016). Mutual information can be used to quantify the mutual information value between each factor and thickness. According to the mutual information value, the importance of each factor can be compared, and then the importance of each factor can be ranked. The larger the mutual information value is, the closer the variable relationship is. The definition of mutual information between two random variables *X* and *Y* is shown in Eq. (15).

(15) \begin{eqnarray*}I(X,Y)=\iint \rho (x,y)\log \nolimits \frac{\rho (x,y)}{\rho (x)\rho (y)} dxdy\end{eqnarray*}

where, *ρ*(*x*) is the edge probability density function of *X*, *ρ*(*y*) is the edge probability density function of *Y*. *ρ*(*x,y*) represents the joint probability density function between random variables *X* and *Y*.

Mutual information is used to realize the feature selection of the original factors. The important factors are selected as the features of the input data set of the model. The specific process of feature selection is as follows:

*Step 1*: Calculate the mutual information between the factors and strip thickness according to Eq. (15), as shown in Table 1.

**Table 1:** Mutual information table of influencing factors and strip thickness.

Factors	Sony value	Temperature	Roll speed	Roll gap	Mill current	Mill rolling force
*I*	0.1684	0.1931	0.5052	0.6269	0.6625	0.8239

*Step 2*: According to the principle of mutual information feature extraction (Wang et al., 2022; Yu et al., 2022). Take the threshold *α* = 0.2.

*Step 3*: The factors with mutual information value *I* greater than *α* are selected. The factors selected according to Table 1 include rolling speed, roll gap, rolling current and rolling force.

### Performance analysis of IBCO algorithm

In order to verify the superior performance of the improved border collie optimization algorithm, IBCO algorithm, border collie optimization (BCO) algorithm, whale optimization algorithm (WOA), grey wolf optimization (GWO) algorithm and particle swarm optimization (PSO) algorithm are jointly used for independent repeated experiments on six test functions to compare the optimization performance of the algorithm (Yin et al., 2021). In the experiment, the size of population is set to 30, the maximum number of iterations is set to 250, and each function is tested for 30 times independently. The test function expressions are shown in Table 2.

**Table 2:** Table of test function formula.

Test function	Specific formula
Function *f*_1_	${f}_{1} \left( x \right) ={\mathop{\sum }\nolimits }_{i=1}^{n}{x}_{i}^{2}$
Function *f*_2_	${f}_{2} \left( x \right) ={\mathop{\sum }\nolimits }_{i=1}^{n} \left\vert {x}_{i} \right\vert +{\mathop{\prod }\nolimits }_{i=1}^{n} \left\vert {x}_{i} \right\vert $
Function *f*_3_	${f}_{3}(x)=ma{x}_{i} \left\{ \left\vert {x}_{i} \right\vert ,1\leq i\leq n \right\} $
Function *f*_4_	${f}_{4} \left( x \right) =-20\exp \left( -0.2\sqrt{ \frac{1}{n} }{\mathop{\sum }\nolimits }_{i=1}^{n}{x}_{i}^{2} \right) -\exp \left( \frac{1}{n} {\mathop{\sum }\nolimits }_{i=1}^{n}\cos 2\pi {x}_{i} \right) +20+e$
Function *f*_5_	${f}_{5} \left( x \right) ={ \left[ \frac{1}{500} +{\mathop{\sum }\nolimits }_{j=1}^{25} \frac{1}{j+{\mathop{\sum }\nolimits }_{i=1}^{2}{ \left( {x}_{i}-{a}_{ij} \right) }^{6}} \right] }^{-1}$
Function *f*_6_	${f}_{6} \left( x \right) ={\mathop{\sum }\nolimits }_{i=1}^{11}{ \left[ {a}_{i}- \frac{{x}_{i}-({b}^{2}+{b}_{i}{x}_{2})}{{b}_{i}^{2}+{b}_{i}{x}^{3}+{x}_{4}} \right] }^{2}$

**Figure 9:**
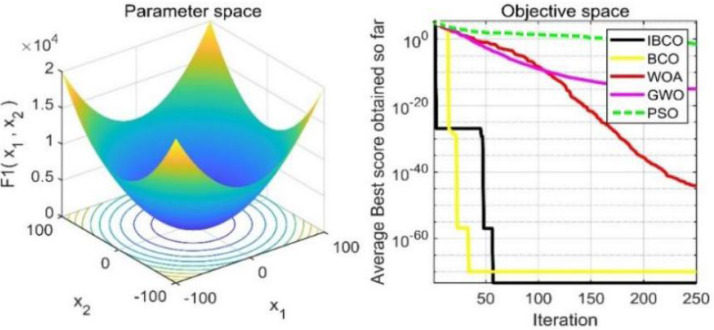
The curve of function f_1_ fitness change.

**Figure 10:**
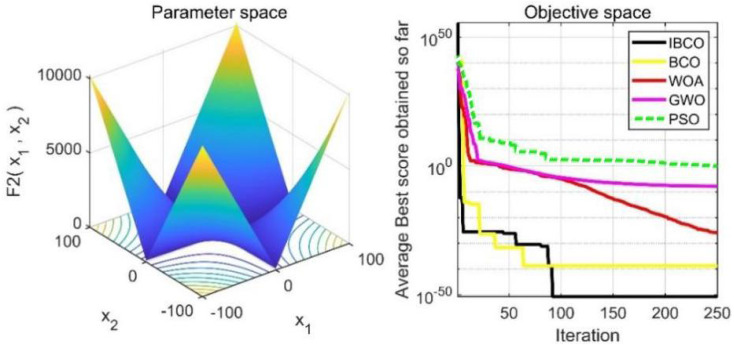
The curve of function f_2_ fitness change.

**Figure 11:**
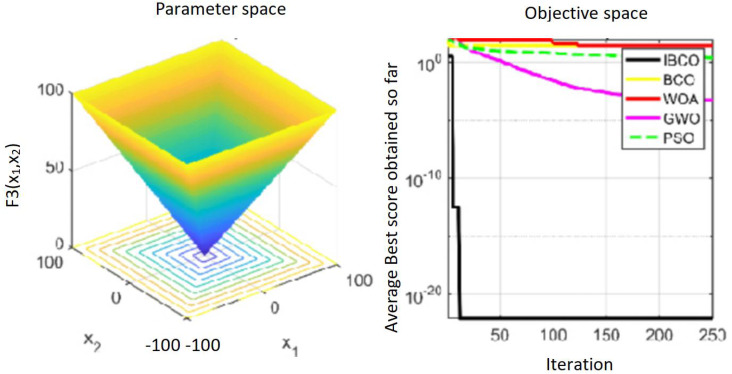
The curve of function f_3_ fitness change.

**Figure 12:**
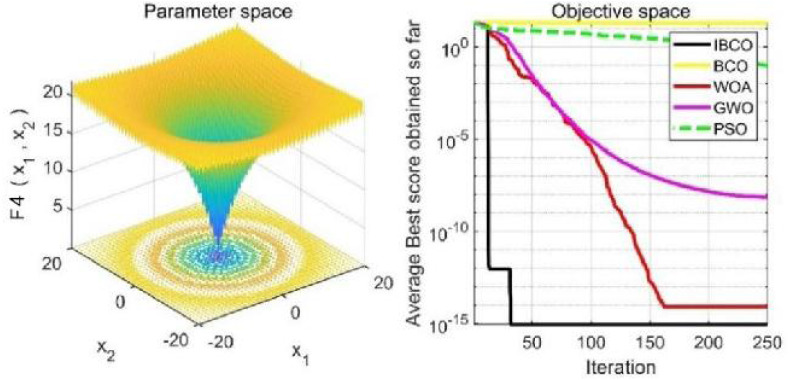
The curve of function f_4_ fitness change.

**Figure 13:**
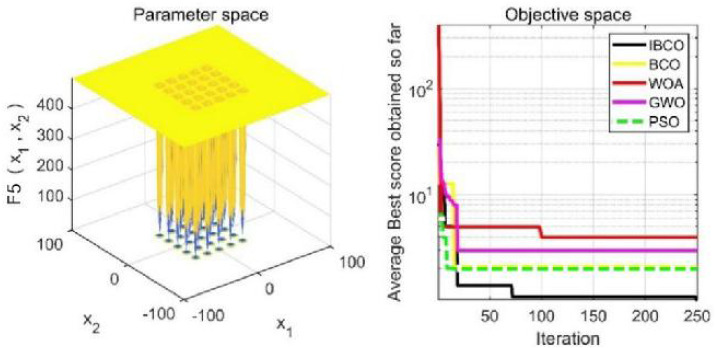
The curve of function f_5_ fitness change.

**Figure 14:**
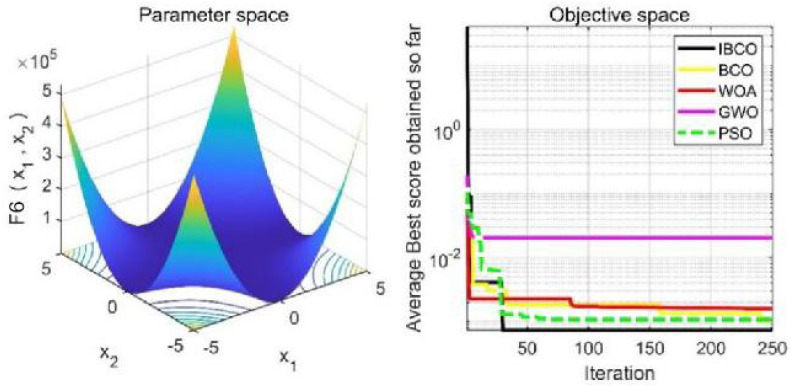
The curve of function f_6_ fitness change.

In Table 2, *f*_1_, *f*_2_ and*f*_3_ are single-peaked test functions, and *f*_4_ is multi-peaked test function. The number of local minimum values of multi-peaked function increases exponentially with the increase of problem dimension, which is the most difficult test function for algorithm optimization. The *f*_5_ and *f*_6_ are the fixed-dimensional multi-peaked test functions, which have only a few local minimums. The optimal values of *f*_1_ to *f*_5_ are 0, and the optimal value of *f*_6_ is 0.0003. The related parameters of each algorithm are initialized: the logarithmic spiral coefficient *b* of the whale algorithm is set to 1, and the search coefficient *a* decreases from 2 to 0. Grey Wolf algorithm collaborative coefficient vector c = [0, 2], and convergence factor a = [0, 2]. The maximum speed of the particle swarm algorithm is set to 6, the inertia weight w = (0.2, 0.9), and the learning factor C1 = C2 = 2. The convergence curves of the five algorithms on six benchmark test functions are shown in Figs. 9 to 14.

Figures 9 to 11 are single-peak function convergence curves. It can be seen that the convergence accuracy of IBCO on function *f*_1_ and *f*_2_ is better than that of BCO, and it is also better than other algorithms. Besides, its convergence effect is obvious. IBCO has the fastest speed of convergence and the highest accuracy on function *f*_3_, and it can still perform well when other algorithms fall into stagnation. Therefore, IBCO has the best ability to find the best when solving for the single-peak function. Figures 12 to 14 show the convergence curves of the multi-peak and fixed multi-peak functions. IBCO can jump out of the local optimum and converge to the optimal value on both the multi-peak function *f*_4_ and fixed multi-peak function *f*_6_ with the highest accuracy of the optimization, and the convergence speed on the function *f*_4_ is very fast.

The algorithm can’t converge to the optimal value of the function *f*_5_, but IBCO still has the best performance and the highest convergence accuracy among the algorithms. The optimization effect of each algorithm on function *f*_5_ is not good and does not converge to the optimal value, but IBCO is still the best in many algorithms and has the highest convergence accuracy. Although the convergence rate of IBCO on function *f*_5_ and *f*_6_ is not the fastest, it converges to a better value at the expense of a little convergence rate and it is generally acceptable. In short, IBCO can jump out of local optimum in multi-peak and fixed multi-peak function. In addition, the optimization accuracy is better than other algorithms and the speed is faster. In order to further analyze the performance of IBCO, the optimal value, average value and standard deviation of each algorithm are compared in Table 3. The optimal value and average value measure the optimization accuracy of the algorithm, and the standard deviation is used to measure the robustness and stability of the algorithm. It can be seen from the table that the optimal value, average value and standard deviation of IBCO in functions *f*_1_, *f*_2_ and*f*_4_ are the lowest, indicating that IBCO has the highest optimization accuracy, the strongest stability and robustness. In functions *f*_3_ and *f*_6_, the optimal value of IBCO is the lowest and the optimization accuracy is the highest standard deviation are at the medium level but better than BCO.

**Table 3:** Test results comparison table of five algorithms.

Test function	Algorithms	Optimum	Average	Standard deviation
*f*_1_(*x*)	IBCO	**5.9121e−107**	**2.577492e−96**	**1.41175e−96**
	BCO	3.4073e−80	2.600108e−75	1.38734e−74
	WOA	4.84e−42	1.559564e−40	6.136066e−40
	GWO	9.9565e−15	9.830024e−15	2.056833e−14
	PSO	0.005012814046	0.01493904	0.01365109
*f*_2_(*x*)	IBCO	**2.1615e−51**	**3.858876e−51**	**6.683491e−50**
	BCO	2.0077e−39	1.07071e−38	1.144189e−38
	WOA	9.8105e−27	3.275149e−27	5.659799e−27
	GWO	1.5076e−08	3.616921e−08	2.99297e−08
	PSO	1.079889073	36.26523	45.93333
*f*_3_(*x*)	IBCO	**8.2795e−23**	0.786683	0.023779
	BCO	26.3125	42.09241	18.42621
	WOA	26.9846	68.60237	24.04321
	GWO	0.00053351	**0.0004998922**	**0.0001614484**
	PSO	2.400611101	1.718419	0.4211281
*f*_4_(*x*)	IBCO	**8.8818e−16**	**8.881784e−16**	**0**
	BCO	1.99991	1.6666361	0.546481
	WOA	7.9936e−15	6.809368e−15	2.05116e−15
	GWO	7.2383e−09	1.114221e−08	4.52399e−09
	PSO	0.09555346357	0.4457932	0.4781256
*f*_5_(*x*)	IBCO	**1.0244**	**1.090221**	**0.1364509**
	BCO	2.0609	1.65959	0.5617166
	WOA	3.9683	1.988086	1.714872
	GWO	2.9821	2.320738	1.145521
	PSO	1.9920	3.304302	2.272921
*f*_6_(*x*)	IBCO	**0.00073362**	0.001058119	0.0005487052
	BCO	0.0010367	0.001237638	0.0005635634
	WOA	0.0015913	**0.0007540982**	0.0007251614
	GWO	0.020363	0.007156549	0.0114377
	PSO	0.001074390894	0.0009900573	**9.206907e−05**

**Notes.**

The bold style indicates that the current value is the optimal value searched by the optimization algorithm.

### Performance analysis of IBCO-LSTM

The prediction performance of IBCO-LSTM, BCO-LSTM, LSTM, BP, SVM and LSSVM applied by scholars in the field of strip thickness prediction are compared and analyzed through experiments. First, the parameters involved in the algorithm are initialized, the population size *n* = 50, the dimension *d* = 3, and the maximum number of iterations *Max_iterations* = 5.

In the experiments, the training data set contains 800 training samples, and the test data set contains 100 test samples. The root mean square error (RMSE) is adopted as an evaluation index to evaluate the prediction accuracy of the six models (Sun et al., 2022). Each model is repeated five times to obtain the average RMSE. The comparison results of different models are shown in Table 4.

**Table 4:** The comparison results of different models.

	IBCO-LSTM	BCO-LSTM	LSTM	BP	SVM	LSSVM
No.	RMSE	RMSE	RMSE	RMSE	RMSE	RMSE
1	0.0054277	0.0071203	0.0087389	0.026182	0.019746	0.017528
2	0.0066768	0.0073653	0.0082481	0.018319	0.020799	0.018471
3	0.0062486	0.0075435	0.0082161	0.01895	0.020116	0.019123
4	0.0059155	0.0074308	0.0079511	0.036869	0.01978	0.017598
5	0.0068407	0.0071089	0.0082362	0.0234132	0.016647	0.018955
Average	0.00622186	0.00731376	0.0082781	0.0247466	0.019428	0.018335

**Figure 15:**
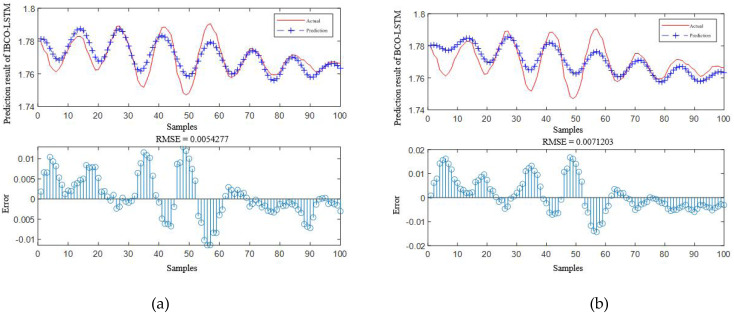
Prediction curve of (A) IBCO-LSTM and (B) BCO-LSTM.

**Figure 16:**
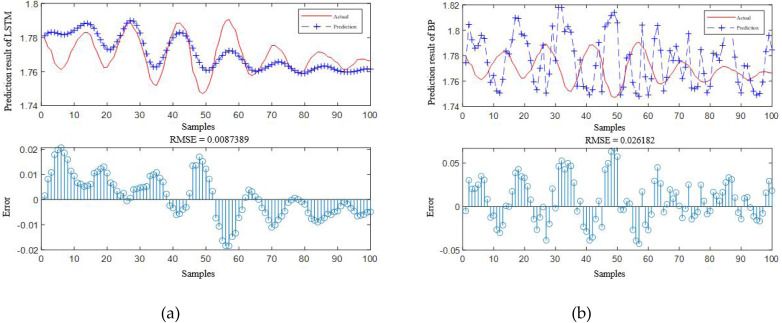
Prediction curve of (A) LSTM and (B) BP.

**Figure 17:**
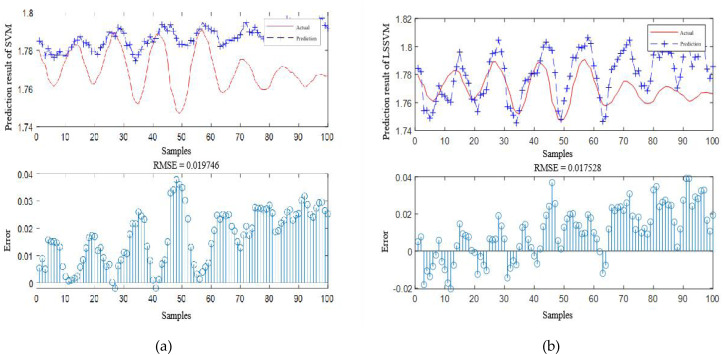
Prediction curve of (A) SVM and (B) LSSVM.

According to Table 4, it is obvious that the prediction accuracy of IBCO-LSTM is higher than those of BCO-LSTM, LSTM, BP, SVM and LSSVM in terms of RMSE and IBCO-LSTM possesses the highest prediction accuracy. In order to visualize the superiority of the prediction performance of the proposed model, the error comparison curves between the prediction results of the six models and the actual values are plotted, as shown in Figs. 15 to 17:

According to the standard of GB709-88, the strip with high rolling accuracy between 1.60 mm and 2.00 mm in strip thickness has the thickness allowable deviation of ±0.13 mm. In Fig. 15, it can be directly seen that the error between the predicted thickness and the actual thickness of IBCO-LSTM is within ±0.02 mm, which is much lower than 0.13 mm. Therefore, the proposed IBCO-LSTM prediction model can meet the actual demand in the rolling field, and the prediction effect is good. If this algorithm is applied to the control system, the rolled strip of the control system can reach the qualified standard.

**Figure 18:**
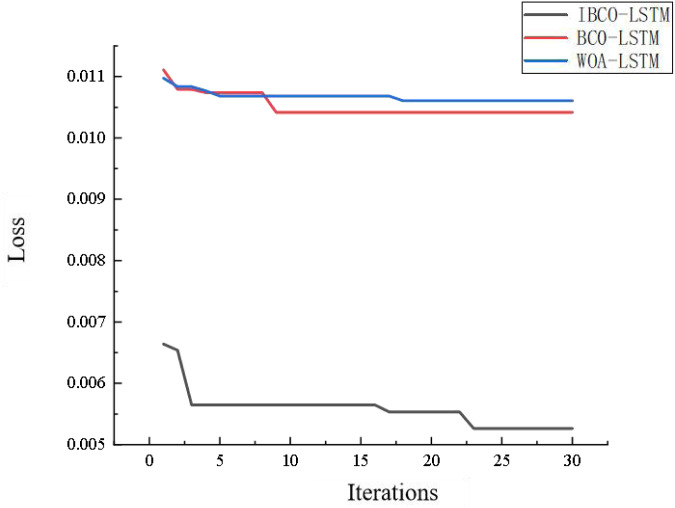
Convergence comparison of models.

Figure 18 is the comparison diagram of the convergence curve of IBCO-LSTM, BCO-LSTM and WOA-LSTM after 30 iterations. From the diagram, it can be seen that IBCO-LSTM has the highest convergence accuracy, namely the lowest prediction error, which indicates that its prediction effect is the best.

## Conclusions

In this article, IBCO-LSTM strip thickness prediction model is proposed to accurately predict the strip thickness. The prediction accuracy of this model is further improved compared with the traditional model, which contributes to the high quality strip in the rolling industry, and the main reasons are as follows:

(1) Due to the large number of data features collected from the actual rolling environment and the nonlinear correlation of all features with strip thickness, mutual information is introduced for feature selection of the data set to reduce the model complexity.
(2) In addition, the prediction accuracy of LSTM is greatly affected by the parameter setting. Therefore, the swarm intelligence algorithm is used to optimize the LSTM parameters, search the optimal LSTM parameters. And the optimal prediction model is constructed to improve the prediction accuracy of strip thickness.
(3) The swarm intelligence algorithm referred in this article is the border collie optimization algorithm, which has strong optimization ability. However, there are problems such as uneven distribution of initial population and inaccurate motion state of some individuals, which affect the convergence accuracy of the algorithm. Therefore, an improved border collie optimization algorithm is proposed. Tent mapping is used to optimize the population initialization method, improve the uniformity and ergodicity of the initial population distribution, and further improve the global search ability of the algorithm. The dynamic weight is introduced into some individual speed updating methods, and the dynamic weighting strategy is used to make the motion state more accurate and improve the local optimization accuracy of the algorithm.

According to a series of comparative experiments, the superiority of the proposed model is verified. For further development, IBCO-LSTM has higher prediction accuracy than some traditional strip thickness prediction models, but more relevant factors may be taken into account if it is to be applied to the complex strip rolling environment, and more in-depth research is needed, such as the coupling between rolling parameters and the prediction process.

##  Supplemental Information

10.7717/peerj-cs.1114/supp-1 Supplemental Information 1 Strip thickness data set, including training set and test set Each row in the data set includes four characteristic parameters of rolling force, roll gap, rolling speed and click power, and the predicted target strip thickness.

10.7717/peerj-cs.1114/supp-2 Supplemental Information 2 Matlab implementation code and comparative experimental code of the method proposed in the article

## Additional Information and Declarations

### Data Availability

The following information was supplied regarding data availability: The raw data are available in the Supplementary Files.
